# Supplementary material for: Putting self at stake by telling a story: Storyteller’s narcissistic traits modulate physiological emotional reactions to recipient’s disengagement
Source: PLoS One. 2024 Aug 27;19(8):e0302703. doi: 10.1371/journal.pone.0302703 (PMC11349197; doi:10.1371/journal.pone.0302703)
Supplement: S1 File — (DOCX) [file pone.0302703.s001.docx]

## Supplementary material


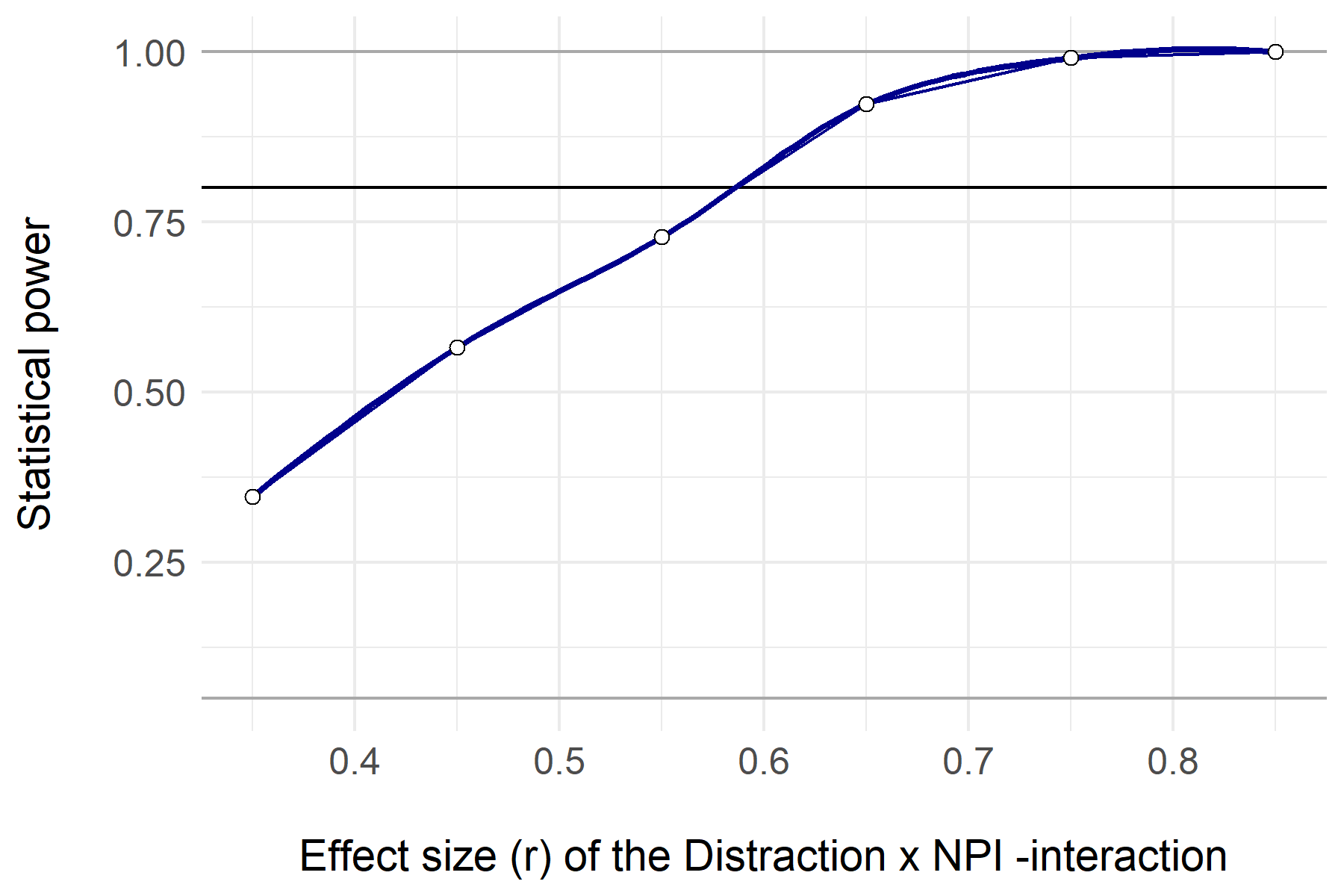


*Supplementary Figure 1.* Power curve for the distraction x NPI -interaction. The power estimates were calculated performing 6000 simulations of the same linear regression model with Distraction condition (x1), NPI13 (x2) and the interaction between the two (x1*x2) as predictors. The power_interaction() function of InteractionPoweR package in R was used. The parameter values were defined as follows: alpha = 0.05, N = 20, r.x1x2.y = seq(.35, .85, by =.10), r.x1.y = -.09, r.x2.y = -.45, r.x1.x2 = .15, k.x1 = 2. The correlations between x1 and y, x2 and y, and between x1 and x2 were based on the acquired data.

*Supplementary Table 1. Regression results using teller’s performance, story potential and listener affiliation as outcomes.*

|  | Teller’s Performance | | |  | Story Potential | | |  | Listener Affiliation | | |
| --- | --- | --- | --- | --- | --- | --- | --- | --- | --- | --- | --- |
| Predictors | b | beta | b  95% CI  [LL, UL] |  | b | beta | b  95% CI  [LL, UL] |  | b | beta | b  95% CI  [LL, UL] |
| Intercept | 3.17 ^***^ |  | 1.72 – 4.62 |  | 3.08 ^***^ |  | 1.47 – 4.69 |  | 3.37 ^**^ |  | 1.35 – 5.39 |
| Age (Teller) | 0.02 | 0.23 | -0.01 – 0.04 |  | 0.02 | 0.16 | -0.01 – 0.04 |  | -0.02 | -0.15 | -0.05 – 0.02 |
| Age (Listener) | -0.01 | -0.15 | -0.04 – 0.01 |  | -0.02 | -0.24 | -0.05 – 0.01 |  | -0.01 | -0.08 | -0.04 – 0.03 |
| Gender (Dyad) | -0.14 | -0.08 | -0.68 – 0.41 |  | -0.24 | -0.12 | -0.85 – 0.36 |  | 0.13 | 0.06 | -0.63 – 0.89 |
| Story length (min) | 0.16 | 0.25 | -0.06 – 0.37 |  | 0.28 ^*^ | 0.38 | 0.04 – 0.52 |  | 0.2 | 0.25 | -0.09 – 0.50 |
| Distraction vs. Control | -0.11 | -0.08 | -0.59 – 0.36 |  | -0.39 | -0.22 | -0.92 – 0.14 |  | -0.04 | -0.02 | -0.70 – 0.62 |
| R^2^ | 0.173 | | |  | 0.283 | | |  | 0.081 | | |

*Note.* *A significant b-weight indicates that the beta-weight is also significant. b represents unstandardized regression weights whereas beta indicates standardized regression weights. LL and UL represent the lower and upper limits of 95% confidence intervals of unstandardized regression weights. * p<0.05 ** p<0.01 *** p<0.001.*

*Supplementary Table 2. Regression results using SAM valence, arousal, and dominance ratings as outcomes (N= 42).*

|  | SAM Valence | | |  | SAM Arousal | | |  | SAM Dominance | | |
| --- | --- | --- | --- | --- | --- | --- | --- | --- | --- | --- | --- |
| Predictors | b | beta | b  95% CI  [LL, UU] |  | b | beta | b  95% CI  [LL, UU] |  | b | beta | b  95% CI  [LL, UU] |
| Intercept | 7.69 ^***^ |  | 5.25 – 10.13 |  | 0.52 |  | -2.80 – 3.85 |  | 6.67 ^***^ |  | 4.01 – 9.33 |
| Age (Teller) | 0.04 | 0.26 | -0.01 – 0.08 |  | -0.02 | -0.11 | -0.08 – 0.04 |  | -0.01 | -0.07 | -0.06 – 0.04 |
| Age (Listener) | -0.03 | -0.2 | -0.07 – 0.02 |  | 0.03 | 0.19 | -0.02 – 0.09 |  | -0.03 | -0.21 | -0.07 – 0.02 |
| Gender (Dyad) | -0.32 | -0.12 | -1.24 – 0.60 |  | 0.93 | 0.25 | -0.32 – 2.18 |  | -0.81 | -0.28 | -1.81 – 0.19 |
| Story length (min) | 0.08 | 0.08 | -0.28 – 0.44 |  | 0.09 | 0.06 | -0.40 – 0.57 |  | 0.36 | 0.32 | -0.03 – 0.74 |
| Distraction vs. Control | -0.36 | -0.15 | -1.16 – 0.44 |  | 1.21 ^*^ | 0.35 | 0.12 – 2.30 |  | -0.34 | -0.13 | -1.21 – 0.53 |
| R^2^ | 0.148 | | |  | 0.197 | | |  | 0.146 | | |

*Note. A significant b-weight indicates that the beta-weight is also significant. b represents unstandardized regression weights whereas beta indicates standardized regression weights. LL and UL represent the lower and upper limits of 95% confidence intervals of unstandardized regression weights. * p<0.05 ** p<0.01 *** p<0.001.*

*Supplementary Table 3. Regression models predicting tellers’ phasic skin conductance (SC) and hear-rate (HR) during the story telling (N=39).*

|  | **Phasic SC (baseline corrected)** | | |  | **HR (baseline corrected)** | | |
| --- | --- | --- | --- | --- | --- | --- | --- |
| Predictors | b | beta | b  95% CI  [LL, UU] |  | b | std. Beta | b  95% CI  [LL, UU] |
| Intercept | 2.53 | 0 | -7.37 – 12.43 |  | 45.47 ^***^ | 0 | 23.55 – 67.40 |
| Age (Teller) | 0.01 | 0.03 | -0.16 – 0.19 |  | -0.1 | -0.09 | -0.47 – 0.27 |
| Age (Listener) | 0.08 | 0.15 | -0.11 – 0.27 |  | -0.61 ^**^ | -0.48 | -1.03 – -0.18 |
| Gender (Dyad) | -1.14 | -0.12 | -4.72 – 2.45 |  | -4.75 | -0.21 | -12.68 – 3.17 |
| Story length (min) | 0.76 | 0.19 | -0.76 – 2.28 |  | -0.12 | -0.01 | -3.38 – 3.13 |
| Distraction | -1.49 | -0.16 | -4.60 – 1.62 |  | -1.46 | -0.07 | -8.11 – 5.18 |
| R^2^ | 0.088 | | |  | 0.221 | | |

*Note. Phasic SC refers to task-related phasic skin conductance activity divided by baseline-related phasic skin conductance activity. HR refers to heart rate (beats per minute) change relative to the baseline HR level. A significant b-weight indicates that the beta-weight is also significant. b represents unstandardized regression weights whereas beta indicates standardized regression weights. LL and UL represent the lower and upper limits of 95% confidence intervals of unstandardized regression weights. * p<0.05 ** p<0.01 *** p<0.001.*

*Supplementary Table 4. Regression model predicting teller’s performance with NPI score as moderator (N=42).*

|  | **Teller’s Performance** | | |
| --- | --- | --- | --- |
| Predictors | b | beta | b 95% CI [LL, UU] |
| Intercept | 2.99 ^***^ | 0.04 | 1.48 – 4.50 |
| Age (Teller) | 0.02 | 0.23 | -0.01 – 0.05 |
| Age (Listener) | -0.01 | -0.16 | -0.04 – 0.01 |
| Gender (Dyad) | -0.09 | -0.05 | -0.64 – 0.47 |
| Story length (min) | 0.21 | 0.34 | -0.03 – 0.45 |
| Distraction | -0.08 | -0.05 | -0.58 – 0.42 |
| NPI | 0.09 | -0.09 | -0.42 – 0.60 |
| Distraction*NPI | -0.30 | -0.20 | -0.86 – 0.27 |
| R^2^ | 0.218 | | |

*Note. A significant b-weight indicates that the beta-weight is also significant. b represents unstandardized regression weights whereas beta indicates standardized regression weights. LL and UL represent the lower and upper limits of 95% confidence intervals of unstandardized regression weights. * p<0.05 ** p<0.01 *** p<0.001.*

*Supplementary Table 5. Regression model predicting teller’s performance with PNI vulnerability as moderator (N=42).*

|  | **Teller’s Performance** | | |
| --- | --- | --- | --- |
| Predictors | b | beta | b  95% CI  [LL, UU] |
| Intercept | 3.00 ^***^ | -0.03 | 1.60 – 4.41 |
| Age (Teller) | 0.02 | 0.25 | -0.01 – 0.05 |
| Age (Listener) | -0.01 | -0.17 | -0.04 – 0.01 |
| Gender (Dyad) | -0.04 | -0.02 | -0.61 – 0.53 |
| Story length (min) | 0.13 | 0.21 | -0.08 – 0.33 |
| Distraction Condition | -0.01 | -0.01 | -0.48 – 0.45 |
| PNI vulnerability | -0.38 | -0.35 | -0.77 – 0.01 |
| Distraction*PNI vulnerability | 0.23 | 0.15 | -0.28 – 0.74 |
| R^2^ / R^2^ adjusted | 0.278 | | |

*Note. A significant b-weight indicates that the beta-weight is also significant. b represents unstandardized regression weights whereas beta indicates standardized regression weights. LL and UL represent the lower and upper limits of 95% confidence intervals of unstandardized regression weights. * p<0.05 ** p<0.01 *** p<0.001.*

*Supplementary Table 6. Regression models using self-reported valence and arousal ratings as outcomes and teller’s NPI score as moderator (N= 42).*

|  | **Self-reported Valence** | | |  | **Self-reported Arousal** | | |
| --- | --- | --- | --- | --- | --- | --- | --- |
| Predictors | b | beta | b 95% CI [LL, UU] |  | b | beta | b 95% CI [LL, UU] |
| Intercept | 7.31 ^***^ | -0.03 | 4.76 – 9.86 |  | 1.05 | -0.01 | -2.44 – 4.54 |
| Age (Teller) | 0.04 | 0.33 | -0.00 – 0.09 |  | -0.03 | -0.14 | -0.09 – 0.04 |
| Age (Listener) | -0.03 | -0.25 | -0.07 – 0.01 |  | 0.04 | 0.21 | -0.02 – 0.10 |
| Gender (Dyad) | -0.36 | -0.13 | -1.29 – 0.58 |  | 0.88 | 0.23 | -0.40 – 2.16 |
| Story length (min) | 0.18 | 0.17 | -0.22 – 0.58 |  | -0.06 | -0.04 | -0.60 – 0.49 |
| Distraction | -0.19 | -0.08 | -1.04 – 0.66 |  | 1.04 | 0.3 | -0.12 – 2.20 |
| NPI | -0.5 | -0.28 | -1.37 – 0.37 |  | 0.21 | 0.19 | -0.98 – 1.39 |
| Distraction*NPI | 0.29 | 0.12 | -0.66 – 1.24 |  | 0.24 | 0.07 | -1.06 – 1.54 |
| R^2^ | 0.189 | | |  | 0.230 | | |

*Note. A significant b-weight indicates that the beta-weight is also significant. b represents unstandardized regression weights whereas beta indicates standardized regression weights. LL and UL represent the lower and upper limits of 95% confidence intervals of unstandardized regression weights. * p<0.05 ** p<0.01 *** p<0.001.*

*Supplementary Table 7. Regression results using self-reported valence, and arousal ratings as outcomes and teller’s PNI Vulnerability score as moderator (N= 42).*

|  | **Self-reported Valence** | | |  | **Self-reported Arousal** | | |
| --- | --- | --- | --- | --- | --- | --- | --- |
| Predictors | b | beta | b 95% CI [LL, UU] |  | b | beta | b 95% CI [LL, UU] |
| Intercept | 7.48 ^***^ | -0.02 | 5.03 – 9.93 |  | 0.69 | -0.04 | -2.64 – 4.03 |
| Age (Teller) | 0.04 | 0.27 | -0.01 – 0.08 |  | -0.01 | -0.08 | -0.07 – 0.05 |
| Age (Listener) | -0.03 | -0.22 | -0.07 – 0.01 |  | 0.04 | 0.22 | -0.02 – 0.10 |
| Gender (Dyad) | -0.17 | -0.06 | -1.16 – 0.82 |  | 0.53 | 0.14 | -0.82 – 1.88 |
| Story length (min) | 0.04 | 0.04 | -0.31 – 0.40 |  | 0.12 | 0.09 | -0.37 – 0.61 |
| Distraction | -0.24 | -0.1 | -1.05 – 0.57 |  | 1.17 ^*^ | 0.34 | 0.06 – 2.27 |
| NPI | -0.43 | -0.26 | -1.11 – 0.26 |  | -0.14 | 0.13 | -1.08 – 0.79 |
| Distraction*NPI | 0.19 | 0.08 | -0.70 – 1.08 |  | 0.71 | 0.2 | -0.50 – 1.92 |
| R^2^ | 0.205 | | |  | 0.251 | | |

*Note. A significant b-weight indicates that the beta-weight is also significant. b represents unstandardized regression weights whereas beta indicates standardized regression weights. LL and UL represent the lower and upper limits of 95% confidence intervals of unstandardized regression weights. * p<0.05 ** p<0.01 *** p<0.001.*

*Supplementary Table 8. Regression results using tellers’ phasic CS and HR as outcomes and PNI-V score as moderator.*

|  | **Phasic SC** | | |  | **HR** | | |
| --- | --- | --- | --- | --- | --- | --- | --- |
| Predictors | b | beta | b  95% CI  [LL, UU] |  | b | beta | b  95% CI  [LL, UU] |
| Intercept | 2.95 |  | -7.79 – 13.69 |  | 47.86 ^***^ | 0.03 | 27.06 – 68.66 |
| Age (Teller) | 0.02 | 0.04 | -0.17 – 0.20 |  | -0.12 | -0.1 | -0.47 – 0.24 |
| Age (Listener) | 0.07 | 0.13 | -0.14 – 0.28 |  | -0.56 ^**^ | -0.45 | -0.97 – -0.16 |
| Gender (Dyad) | -1.01 | -0.11 | -5.19 – 3.17 |  | -6.77 | -0.3 | -14.98 – 1.45 |
| Story length (min) | 0.56 | 0.15 | -1.07 – 2.19 |  | 0.49 | 0.06 | -2.63 – 3.62 |
| Distraction | -1.28 | -0.14 | -4.67 – 2.11 |  | -3.01 | -0.15 | -9.41 – 3.38 |
| PNI-V | 0.18 | 0.04 | -2.60 – 2.96 |  | 5.37 ^*^ | 0.4 | 0.11 – 10.64 |
| Distraction*PNI-V | 0.04 |  | -3.59 – 3.66 |  | -2.56 | -0.13 | -9.43 – 4.31 |
| Observations | 38 | | |  | 39 | | |
| R^2^ | 0.061 | | |  | 0.352 | | |

*Note. Phasic SC refers to task-related phasic skin conductance activity divided by baseline-related phasic skin conductance activity. HR refers to heart rate (beats per minute) change relative to the baseline HR level. A significant b-weight indicates that the beta-weight is also significant. b represents unstandardized regression weights whereas beta indicates standardized regression weights. LL and UL represent the lower and upper limits of 95% confidence intervals of unstandardized regression weights. The PNI-V stands for standardized vulnerability sub-scale score of pathological narcissism inventory (PNI). * p<0.05 ** p<0.01 *** p<0.001.*
