# Supplementary material for: Putting self at stake by telling a story: Storyteller’s narcissistic traits modulate physiological emotional reactions to recipient’s disengagement
Source: PLoS One. 2024 Aug 27;19(8):e0302703. doi: 10.1371/journal.pone.0302703 (PMC11349197; doi:10.1371/journal.pone.0302703)
Supplement: Attachment — Submitted filename: Response to Reviewers.docx [file pone.0302703.s002.docx]

**PLOS ONE: Response to Reviewers**

Dear Editor and Reviewers of PLOS ONE,

We thank you for the valuable feedback and useful suggestions to improve our manuscript. In this memo we respond to the comments and questions raised to the best of our abilities. Our responses are written in blue.

Reviewer #1:

Notwithstanding, there are a few points the authors may wish to consider in a subsequent revision:

- Being that a key variable in this study is narcissistic pathology, it was somewhat surprising the very brief narcissism measures were used to operationalize this element. As a result, it is reasonable to quite seriously question the robustness of the narcissism variable under examination and any conclusions to be drawn (particularly if there were more significant findings). Further, the two expressions of narcissism were measured using scales that are somewhat contradictory in their theoretical assumptions (i.e., 'healthy/adaptive' and 'pathological' expressions). While there is some discussion of this regarding the use of the Five-Factor-Model trait variants, but I think more could be made of this limitation, which is quite central to the design of the study.

Thank you for this insightful comment. Narcissism research has identified unique, but overlapping dimensions of narcissism (e.g., grandiose and vulnerable narcissism) that incorporate traits and behaviors that can be viewed as respectively adaptive (e.g., grandiosity, high self-esteem, extraversion) and pathological (e.g., vulnerability, low self-esteem, increased psychopathological symptoms). Despite their distinctions, both aspects of grandiose and vulnerable narcissism are related to psychopathological and interpersonal outcomes (e.g., both are related to Narcissistic Personality Disorder (NPD), relationship difficulties, and elevated reactivity to socio-evaluative stressors). Recent research has also noted that these manifestations of narcissism may best be captured using multiple narcissism measures or more recently developed (and less established) multi-factor narcissism measures. Our decision to use the NPI and the PNI was based on a review of previous research demonstrating their respective capabilities to capture the grandiose and vulnerable dimensions. Moreover, brief versions of these measures were recently validated in a Finnish sample (see Henttonen et al., 2022, and comments below). Finally, we wanted to include multiple narcissism dimensions while keeping the total experiment duration to a minimum and keep the ratio of time used for the paradigm and form-filling reasonable. However, we carefully examined and decided on the selected measures prior to the experiment.

To our understanding, the brief versions of NPI (Gentile et al., 2013) and PNI (Schoenleber et al., 2015) have demonstrated convergent and divergent validity comparable to the original versions using adequately sized samples among clinical and community participants. In a separate prior validation study (n=439, Henttonen et al., 2022), using a comparable sample, the translated brief versions of the questionnaires were observed to demonstrate good psychometric properties in general and in relation to other brief measures of narcissism (HSNS, g-FLUX). Affirming previous findings, NPI-13 accounted for the greatest amount of variance in self-reported PDQ-NPD symptoms and conformed to the expected pattern of higher self-esteem and well-being, while SB-PNI Vulnerability exhibited the largest predictive power for diminished self-esteem, well-being and psychopathology.

The references to the original and brief versions of the scales and the Finnish validation study are included in the Method section (p. 10), whereas rationale concerning the contemporary debate on grandiose-vulnerable vs three-facet dimensions of narcissism is included in the discussion section in the original manuscript. However, we have added an additional statement to the limitations section (page 18): “due to experimental constraints, brief versions of the measures were used to operationalize narcissism. Usage of full-length versions and/or additional scale instruments could potentially increase the psychometric accuracy in measuring the underlying trait(s) of interest.”

- A similar limitation relates to the sample under examination being university students. While quite a common convenience sample to select, it is not ideal for examining narcissistic pathology as scores in published literature using such samples tend to be quite low - which again raises question to what extent are we truly examining narcissistic pathology. Again, there is a comment on this in the limitations, however this could be expanded. While of course nothing can be done in the current design to account for this, the authors could comment on possible experimental designs that would allow for a more robust examination, using clinical samples and more rigorous narcissism and psychopathological constructs to help guide future research which may like to build on this study.

In this study we adopted a continuum view of narcissism where individuals are seen to exhibit traits of grandiose and vulnerable narcissism in varying degrees, ranging from low levels to pathological levels. According to this view, studying participants with relatively low levels of these traits can still provide insight on the phenomenon of narcissism in general. We have also tried to select our scale instruments carefully in order to also include pathological aspects of narcissism (see the comment above). However, as there is also evidence that while grandiose and vulnerable narcissism are unrelated at low-to-moderate levels of grandiose narcissism, they are related at high levels of grandiose narcissism (Jauk et al. 2022) and thus there can be special physiological patterns regarding individuals with clinically relevant narcissism. We have now expanded the limitations section to include this information, as well as made suggestions for future studies according to the reviewer’s suggestion (see page 19).

- Finally, while some participant demographics and measures descriptives are variously included in text throughout, the manuscript would benefit from a table summarizing these features in order to assist readers to understand the sample under examination better.

We have included full information of the participant demographics, per condition and role, regarding age and NPI/PN sum scores in the new Table 1 (page 7). The table also depicts the experimental measures, per condition, of rated story quality, teller performance, recipient affiliation, PANAS valence, arousal and dominance. Other table identifiers have been modified accordingly.
